# O064. Antiepileptic drugs in migraine and epilepsy disorders: who is at increased risk of adverse events?

**DOI:** 10.1186/1129-2377-16-S1-A69

**Published:** 2015-09-28

**Authors:** Michele Romoli, Sabrina Siliquini, Ilenia Corbelli, Stefano Caproni, Chiara Bedetti, Laura Bernetti, Elona Brahimi, Cinzia Costa, Paola Sarchielli, Paolo Calabresi

**Affiliations:** Neurology Clinic, Perugia University Hospital, Perugia, Italy; IRCCS Fondazione “S. Lucia”, Rome, Italy

## Background

Migraine and epilepsy are chronic disorders, often comorbid, characterized by transient and recurrent neurological disturbances. Sharing pathophysiologic and clinical features, both epilepsy and migraine benefit from antiepileptic drugs (AEDs) treatment. However, despite their overlapping, peculiar differences regarding reported adverse events (AEs) of AEDs seem to emerge in clinical practice. In particular, tolerability and frequency of AEs might depend on the condition from which the patient suffers. Therefore, we interviewed patients treated with AEDs for epilepsy, migraine, and both, in order to compare AEs distribution of frequency among the three groups.

## Materials and methods

We collected AEs of some AEDs - valproic acid (VPA), topiramate (TPM), lamotrigine (LTG) - widely used in prophylactic therapy of migraine, in epilepsy as well as in epileptic migraineurs. All AEs were gathered through the Liverpool Adverse Events Profile (LAEP)[1].

## Results

Three hundred and thirty-five patients were recruited: 142 suffered from epilepsy (group A), 131 from headache (group B), 62 from both (group C). Mean age was 44.5 in group A, 45.0 in B, 40.5 in C. AEs were significantly more reported in group B (69.5%) and under TPM treatment (71%). The most prescribed AED for group B was TPM, which was more commonly referred to cause paresthesias (68%) and language disorders (42%) among this group than in the other two. Complaints of weight gain were common with VPA in all three groups, with higher frequencies among group B and C. Memory impairment induced by AEDs was reported more frequently for TPM in all three groups, while maximal incidence was reported for VPA and TPM, respectively in group B (5%) and C (9%). Overall, migraineurs were more likely to drop out of treatment (46%) than epileptic patients (29.6%) and patients with epilepsy and migraine (41.9%).

## Discussion and conclusions

Our data confirm the extensive safety and effectiveness of AEDs in clinical practice, and point to patient's tolerability of AEs as pivotal for a successful treatment[2]. We emphasize the higher prevalence of AEs due to AEDs in migraineurs, suggesting a peculiar susceptibility of their condition to experience AEs. This finding, which emerges despite the average higher dosage of AED used for epilepsy, remains actually unexplained. Our results might be intriguingly considered as a clinical implication of central sensitization mechanisms or, not less intriguingly, they might represent the result of an abnormal network plasticity producing microstructural changes in migraine-affected brain[3].

Written informed consent to publication was obtained from the patient(s).
